# Mining for phosphates: the *Myb73-1*-*GDPD2*-*GA2ox1* module as a hub to improve phosphate deficiency tolerance in soybean

**DOI:** 10.1093/plcell/koae056

**Published:** 2024-02-20

**Authors:** Christian Damian Lorenzo

**Affiliations:** Assistant Features Editor, The Plant Cell, American Society of Plant Biologists; Center for Plant Systems Biology, VIB, B-9052 Ghent, Belgium; Department of Plant Biotechnology and Bioinformatics, Ghent University, B-9052 Ghent, Belgium

A highly plastic root architecture is critical for nutrient uptake to support plant growth and development. Lacking the capacity to move, plants adjust their root systems to reach vital elements such as nitrogen (N) and phosphorus (P). Soybeans and other legumes—important sources of proteins and oils for human food and animal feed—require little to no N fertilizer, owing to their capacity for N_2_ fixation. However, this process is strongly reliant on P nutrition (Zhong et al. 2023). In addition, no more than 30% of applied P in fertilizers can be taken up by crops in the form of phosphate (Pi) (López-Arredondo et al. 2014), which makes this macronutrient limiting in modern agriculture. Therefore, expanding our understanding of Pi uptake in crops is pivotal to developing new varieties that can better mine this essential mineral.

In this issue of *The Plant Cell*, **Hu and colleagues (Hu et al. 2024)** identified a regulatory module composed of 3 genes controlling low-Pi–associated traits (Fig.). The authors focused on characterizing genes regulating root surface traits, aiming for enhancements that result in more efficient Pi acquisition. By integrating linkage mapping and genome-wide association studies (GWAS) data in a diverse soybean panel with varying Pi uptake efficiency, they further analyzed a previously identified quantitative trait locus (QTL), qPE19, positively associated with beneficial root traits (Zhang et al. 2010). Digging further into the genomic region of qPE19, they detected a gene encoding a glycerophosphoryl diesterphosphodiesterase, *GmGDPD2*, with high expression in roots in low Pi (LP) vs normal Pi (NP) treatments. By analyzing natural variation in the promoter and coding regions, they observed that highly Pi-efficient cultivars presented variations that lead to higher expression of *GmGDPD2*. Employing both overexpressor (OE) and CRISPR mutant knockout (KO) lines, they directly linked *GmGDPD2* expression levels to increases and decreases in LP root traits under LP and NP treatment experiments. Furthermore, by studying the root transcriptomes of *GmGDPD2* OE and KO lines, they found several genes connected to root traits that were differentially expressed, such as acid phosphatases, Pi transporters, and DELLA-type genes.

**Figure.**
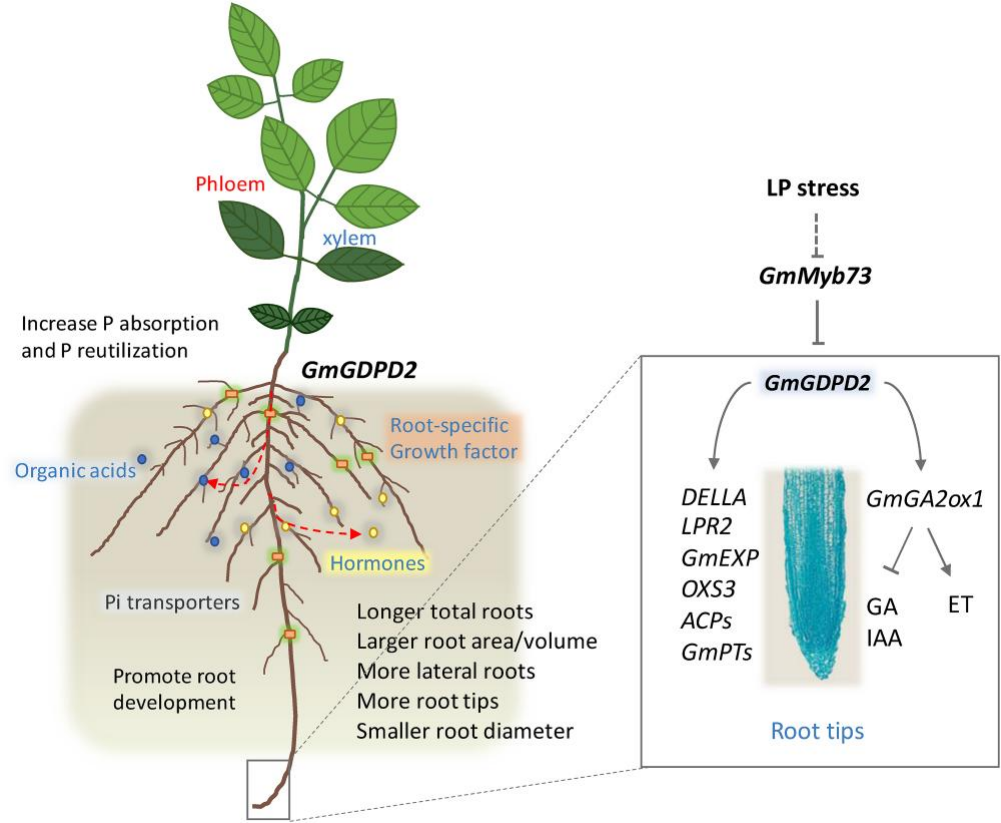
Proposed model for the *GmGDPD2* hub in Pi tolerance in soybean. Upon LP stress, *GmMyb73* repression over *GmGDPD2* is released, reducing gibberellins (GAs) and auxins in root tips via GmGA20 × 1 action. Alongside altered expression of other PI-responsive genes, root architecture is modified to enhance Pi uptake. Reprinted from Hu et al. (2024), Fig. 7.

Knowing that *GmGDPD2* plays an important role in LP uptake, the authors sought to uncover the associated gene network. Using yeast 1-hybrid and luciferase reporter assays, they identified an R2R3-type transcription factor, GmMyb73, which binds to the *GmGDPD2* promoter. *GmMyb73* presented the opposite gene expression to Pi treatments compared to *GmGDPD2* (high in NP and low in LP), as well as contrasting phenotypes in OE and silenced lines (RI) to that of *GmGDPD2* OE and KO plants. Another module element, *gibberellin 2-dioxygenase* (*GmGA2ox1*), was identified by testing direct protein interactions with *GmGDPD2* through double hybrid assays. Linked to the upregulation of active GA and auxin levels, *GmGA2ox1* expression was downregulated and upregulated in KO- and OE-*GmGDPD2*, respectively, and *GmGA2ox1* OE and KO lines displayed similar (but milder) increases in root traits to *GmGDPD2* lines. Moreover, upon stacking with the *GmGDPD2* KO, the double mutant *KO-GmGDPD2GmGA2ox1* presented further reductions in all root parameters and Pi uptake compared to single mutants under both NP and LP conditions. All these data suggest a synergistic role of both genes in LP tolerance traits.

Finally, the authors assessed the breeding value of elements of this module by phenotyping soybean yield traits. They observed that *GmGDPD2* OE lines presented significant increases in seed yield, branch number, pod number, protein content, and seed weight. In contrast, KO *GmGDPD2* lines suffered significant penalties in all yield traits evaluated, supporting the importance of *GmGDPD2* for increased performance. Integrating all the data obtained, the researchers built a model to explain how LP tolerance is regulated by a *GmGDPD2*-centered module (Fig.). Upon Pi starvation, the expression of *GmGDPD2* (constrained by *GmMyb73* under NP) rises and results in a modified root architecture through *GmGA2ox1* activity. *GmGA2ox1* reduces GA levels and thus inhibits root elongation, resulting in a remobilization of auxin from root tips, which induces the formation of lateral roots and root hairs that affect Pi uptake and/or Pi translocation. Thus the *GmGDPD2* module represents a promising hub that could be exploited to enhance soybean LP stress tolerance by remodeling root architecture traits.
